# Multiple Primary Malignancy-Related Gallbladder Cancer: An Important Recommendation for the Early Detection of Gallbladder Cancer

**DOI:** 10.3390/diagnostics16040605

**Published:** 2026-02-19

**Authors:** Hiroko Naganuma, Hideaki Ishida, Naoki Matsumoto, Masahiro Ogawa

**Affiliations:** 1 Department of Gastroenterology, Yokote Municipal Hospital, Akita 013-8602, Japan; 2 Department of Gastroenterology, Akita Red Cross Hospital, Akita 010-1495, Japan; minnnanous@gmail.com; 3 Division of Gastroenterology and Hepatology, Department of Medicine, Nihon University School of Medicine, Tokyo 101-8309, Japan; matsumoto.naoki@nihon-u.ac.jp (N.M.); echo.m.ogawa0922@gmail.com (M.O.)

**Keywords:** gallbladder cancer, multiple primary malignancies, contrast-enhanced ultrasonography, risk factor, postoperative surveillance system

## Abstract

**Background**: Gallbladder cancer (GBC) is a highly lethal malignancy that is often asymptomatic in its early stages and difficult to treat once clinical symptoms appear. Although established risk factors include female sex, gallstones, and chronic biliary inflammation, other clinical contexts associated with GBC remain insufficiently characterized. Multiple primary malignancies (MPMs), in which more than one primary cancer develops in the same individual, have recently attracted attention; however, their relationship with GBC has only rarely been examined. In this study, we aimed to clarify the clinical features of MPM-related GBC and explore its implications for early detection. **Methods**: We retrospectively compared 22 patients with GBC associated with other malignancies (MPM-positive GBC) and 16 patients with GBC alone (MPM-negative GBC). Clinical characteristics, major risk factors, tumor presentation, and imaging findings were evaluated. Special attention was paid to the sequence of cancer development and the diagnostic utility of contrast-enhanced ultrasonography (CEUS). **Results**: In all MPM-positive cases, GBC occurred as a second primary malignancy, most commonly following gastrointestinal cancers (12/22). Gallstones were significantly less frequent in the MPM-positive group than in the MPM-negative group (2/22 vs. 6/16). The MPM-positive group showed a slight male predominance (12 males and 10 females). Neither pancreaticobiliary maljunction nor porcelain gallbladder was identified in either group. CEUS was useful for both the detection and qualitative diagnosis of GBC in all patients. **Conclusions**: MPM-related GBC frequently develops as a second primary malignancy in patients with prior gastrointestinal cancer and may arise in the absence of classical risk factors. Careful long-term surveillance after cancer treatment is therefore essential for identifying second primary GBC at a potentially resectable stage. The combined use of ultrasonography and CEUS may facilitate earlier and more accurate diagnosis in this clinical setting.

## 1. Introduction

Gallbladder cancer (GBC) is a highly lethal neoplasm, with a five-year survival rate of less than 10% [1,2]. It is often asymptomatic in its early stages and becomes difficult to treat once symptoms appear; consequently, substantial improvement in prognosis has yet to be achieved [2,3]. Therefore, early detection remains a critical unmet need. Against this background, several established risk factors for GBC have been reported, including female sex [4], the presence of gallstones [5,6], pancreaticobiliary maljunction (PBM) [7,8], primary sclerosing cholangitis [9], porcelain gallbladder [10], and others [11,12].

Meanwhile, an increasing amount of attention is being directed toward the phenomenon in which multiple primary cancers occur in the same individual, referred to as multiple primary malignancies (MPMs), with a marked rise in reported cases in recent years [13,14,15,16,17,18,19]. MPMs are defined as the occurrence of two or more primary cancers arising in different organs, either synchronously (diagnosed within six months of each other) or metachronously (diagnosed more than six months apart). Distinct patterns have been described with respect to MPM combinations, such as gastrointestinal–gastrointestinal malignancies or combinations involving non-gastrointestinal organs, and prognosis has been shown to depend largely on the pairing of the first and second primary cancers and whether the presentation is synchronous or metachronous [16,17,18,19]. From this perspective, detailed evaluation of tumor combinations is of considerable clinical importance. However, despite MPMs’ potential relevance to early cancer detection, this issue has been scarcely investigated in the context of gallbladder cancer [20,21].

The growing interest in MPMs can be attributed to several factors. Advances in imaging technologies have facilitated the detection of malignancies at asymptomatic stages, while improvements in the treatment of first primary cancers have prolonged patient survival, thereby increasing the likelihood of developing second primary malignancies. In this clinical context, imaging modalities capable of identifying subtle lesions play an increasingly important role. Although the available diagnostic algorithms for malignant tumors vary among institutions depending on available expertise and resources, ultrasonography—particularly when combined with contrast-enhanced ultrasonography (CEUS)—remains a valuable tool for detecting small or early-stage lesions, including those of the gallbladder [22,23].

In this study, although the number of cases was relatively small (83 cases), we divided gallbladder cancer cases into MPM-positive (MPM (+)) and MPM-negative (MPM (−)) groups and performed a comparative analysis. Through this approach, we aimed to obtain clinically useful insights into the relationship between MPM and gallbladder cancer.

## 2. Materials and Methods

We compared 22 patients with gallbladder cancer who also experienced other malignancies (MPMs (+) GBC) with 16 patients who had gallbladder cancer alone (MPMs (−) GBC). The 22 patients in the MPM (+) GBC group comprised 12 men and 10 women, aged 59–88 years (mean, 75.3 years). The 16 patients in the MPM (−) GBC group comprised 8 men and 8 women, aged 51–86 years (mean, 73.7 years).

The relatively small sample size resulted from our decision to include only surgically resected, histologically confirmed cases in order to ensure diagnostic accuracy. The observation period extended from July 2010 to July 2025, and this retrospective study was conducted across three affiliated hospitals.

The following points were evaluated in both groups (Table 1):

(1) We determined whether the GBC represented the first or second primary malignancy and examined the types of malignancies other than GBC;
(2) We scanned for major risk factors for GBC such as gallstones, pancreaticobiliary maljunction, primary sclerosing cholangitis, or porcelain gallbladder;
(3) We evaluated symptoms at the time of GBC diagnosis and corresponding imaging findings;
(4) We evaluated maximum tumor diameter and depth of invasion based on surgical specimens;
(5) Unresectable GBC cases encountered during the same period were also analyzed, divided into MPM (+) and MPM (−) groups, and compared with respect to points (1)–(3);
(6) Prognostic comparisons were performed among four groups: MPM (+) resected (group a), MPM (+) unresected (group b), MPM (−) resected (group c), and MPM (−) unresected (group d).

For imaging evaluation, findings from contrast-enhanced ultrasonography (CEUS), computed tomography (CT), and magnetic resonance imaging (MRI) were reviewed for patients who underwent these examinations. During CEUS, lesions demonstrating heterogeneous hyperenhancement followed by early washout were considered highly suggestive of GBC. During CT and MRI, enhancing gallbladder wall thickening was regarded as a finding strongly suggestive of GBC; During MRI, this category also included lesions showing diffusion restriction. When gallbladder wall thickening was present without definite enhancement, the finding was classified as detection only. If no lesions could be identified, the case was categorized as non-diagnostic.

In this study, the final diagnosis of unresectable GBC was established based on comprehensive evaluation of medical imaging findings.

Overall survival was analyzed using the Kaplan–Meier method, and differences between groups were assessed with the log-rank test. The endpoint was defined as death from any cause or transfer to another hospital, whichever occurred first. The Cox proportional hazards model was applied to estimate hazard ratios and 95% confidence intervals.

This study was conducted in accordance with the Declaration of Helsinki. Given the retrospective nature of this study, the requirement for written informed consent was waived.

## 3. Results

The results were as follows:

(1) The MPM (+) GBC group showed a male predominance (12 males, 10 females) (59–88 years old, with a mean age of 74), unlike the MPM (−) group (8 males, 8 females) (51–86 years old, with a mean age of 74). The MPM (+) GBC group consisted of 12 synchronous cases (eight males, four females) (59–87 years old, with a mean age of 75) and 10 metachronous cases (five males, five females) (60–88 years old, with a mean age of 76). In synchronous MPM (+) GBC cases, the other primary cancers were colorectal cancer (three cases), esophagogastric cancer (three cases), breast cancer (two cases), and others (four cases). In metachronous MPM (+) GBC cases, GBC represented the second primary malignancy in all cases, and the first primary cancer was most often colorectal cancer (six cases), followed by gastric cancer (two cases) and others (two cases).
(2) Gallstones were observed in only 2 of the 22 MPM (+) cases (9%), while this figure was 6 of 16 (37.5%) in the MPM (−) group; neither group exhibited pancreaticobiliary maljunction, primary sclerosing cholangitis, or porcelain gallbladder, and in our series, there were no heavy smokers or alcohol abusers, although mild diabetes mellitus could not be completely excluded.
(3) Only one of two cystic duct cancer cases in the 22 MPM (+) GBC group presented transitory abdominal pain; the other 21 MPM (+) GBC cases were asymptomatic. In contrast, 16 MPM (−) GBC cases showed frequent abdominal symptoms (7/16: 43.8%), due to gallstone-related acute cholecystitis.

**Figure 1:**
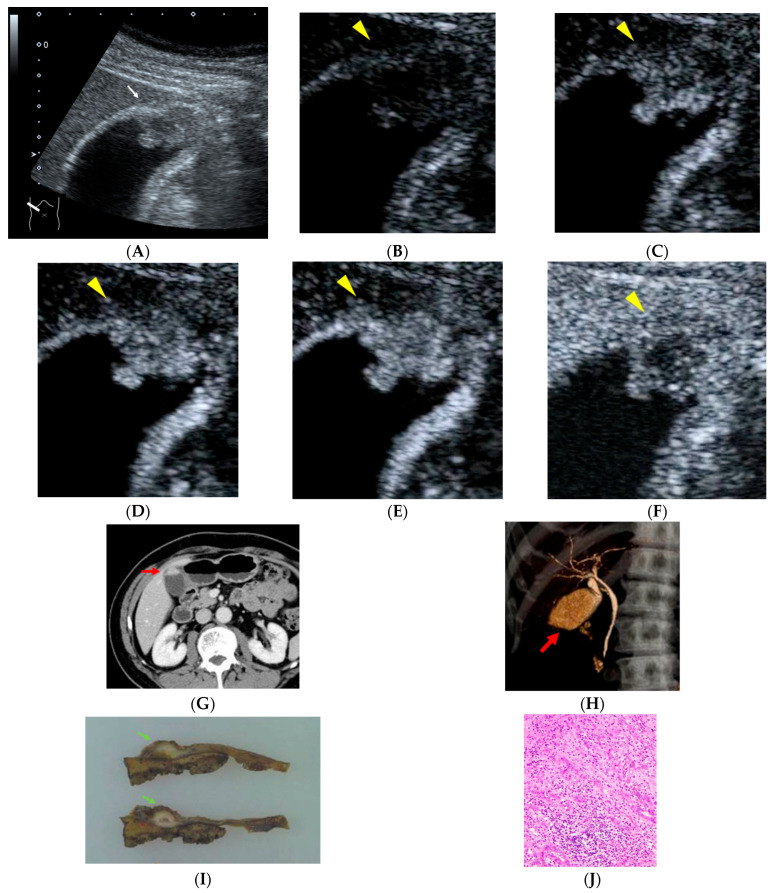
A 66-year-old man with a history of rectal cancer surgery 19 years earlier. During follow-up, a polypoid lesion with a slightly hypoechoic center is detected at the fundus of the gallbladder, accompanied by irregularity of the gallbladder wall ((**A**), white arrow). On contrast-enhanced ultrasonography (CEUS), the polypoid lesion shows heterogeneous enhancement in the early phase followed by washout ((**B**), 0 s; (**C**), 18 s; (**D**), 19 s; (**E**), 22 s; (**F**), 300 s after injection of contrast material, yellow arrowheads). Contrast-enhanced abdominal CT demonstrated an enhancing polypoid lesion at the gallbladder fundus ((**G**), red arrow). CT cholangiography revealed a filling defect at the gallbladder fundus ((**H**), red arrow). Histopathological examination revealed gallbladder carcinoma composed of both well-differentiated and poorly differentiated adenocarcinoma components, with partial invasion into the liver bed, corresponding to stage IIIb disease ((**I**)**,** green arrows; (**J**)). The patient has remained recurrence-free for 65 months after surgery.

**Figure 2:**
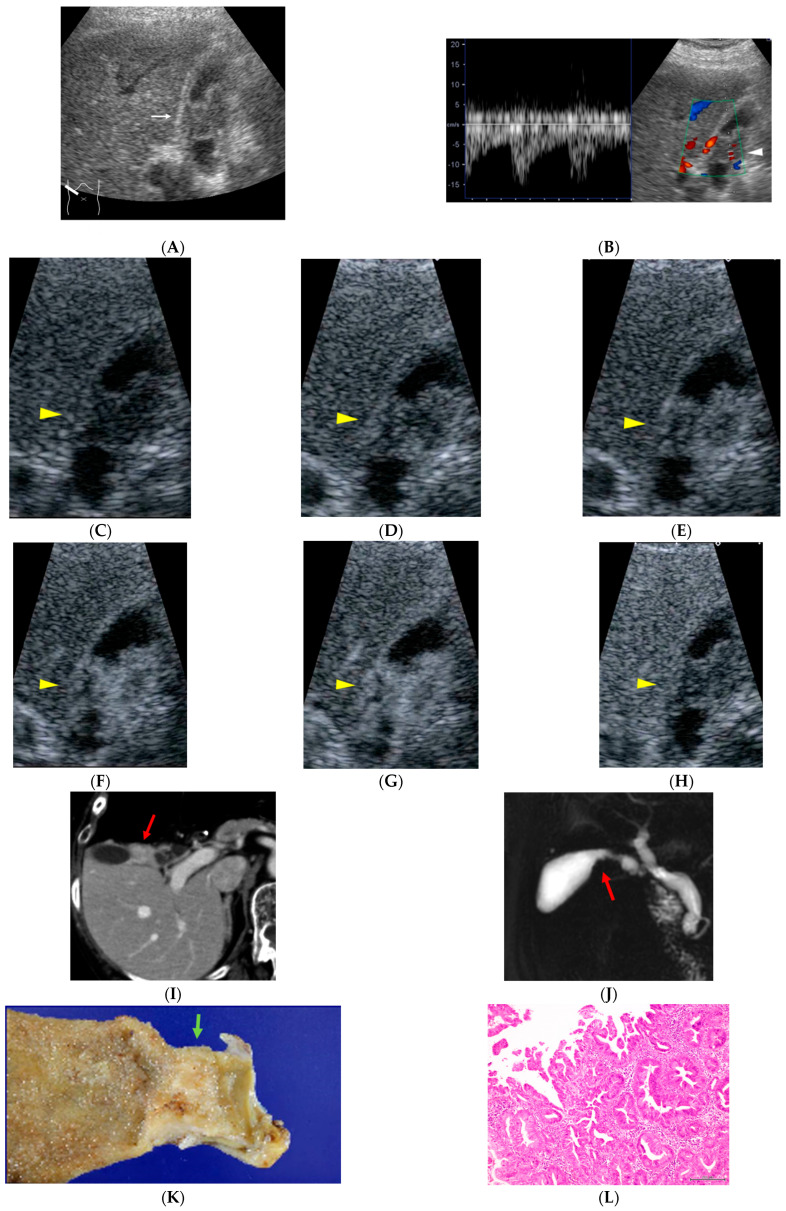
A 77-year-old woman who had been treated for gastric malignant lymphoma 10 years earlier. During evaluation for abdominal pain, diffuse circumferential wall thickening of the gallbladder body is observed ((**A**), white arrow). Color Doppler ultrasonography demonstrated pulsatile intratumoral blood flow signals ((**B**), white arrowhead). On CEUS, the lesion shows early heterogeneous enhancement followed by washout ((**C**), 0: pre-contrast image; (**D**), 15; (**E**), 18; (**F**), 19; (**G**), 22; (**H**), 300 s after injection of contrast material, yellow arrow heads). Contrast-enhanced abdominal CT reveals an enhancing mass lesion at the gallbladder neck ((**I**), red arrow), and MR cholangiopancreatography demonstrates a corresponding filling defect ((**J**), red arrow). Histopathological examination confirms papillary adenocarcinoma, stage II ((**K**), green arrow; (**L**)). The patient remains recurrence-free for 71 months after surgery.

Among the 38 surgically resectable cases, including both the MPM (+) and MPM (−) GBC groups, CEUS was performed for all patients (38/38, 100%). In every case, CEUS demonstrated localized gallbladder wall thickening with early hyperenhancement and early washout. CT was performed for all patients (38/38, 100%). Findings strongly suggestive of GBC were identified in 4 of 38 cases (10%), detection of gallbladder wall thickening without definitive malignant features was achieved in 31 of 38 cases (82%), and CT was non-diagnostic in 3 of 38 cases (8%). MRI was performed in 26 cases. MRI findings were strongly suggestive of GBC in 8 of 26 cases (31%), allowed detection only in 13 of 26 cases (50%), and were non-diagnostic in 5 of 26 cases (19%). Overall, CEUS consistently demonstrated enhancement patterns considered strongly suggestive of malignancy in all resectable GBC cases, whereas CT and MRI more often yielded findings limited to lesion detection or were non-diagnostic, suggesting lower qualitative diagnostic sensitivity for small or early-stage gallbladder lesions (Figure 1 and Figure 2).

**Figure 3:**
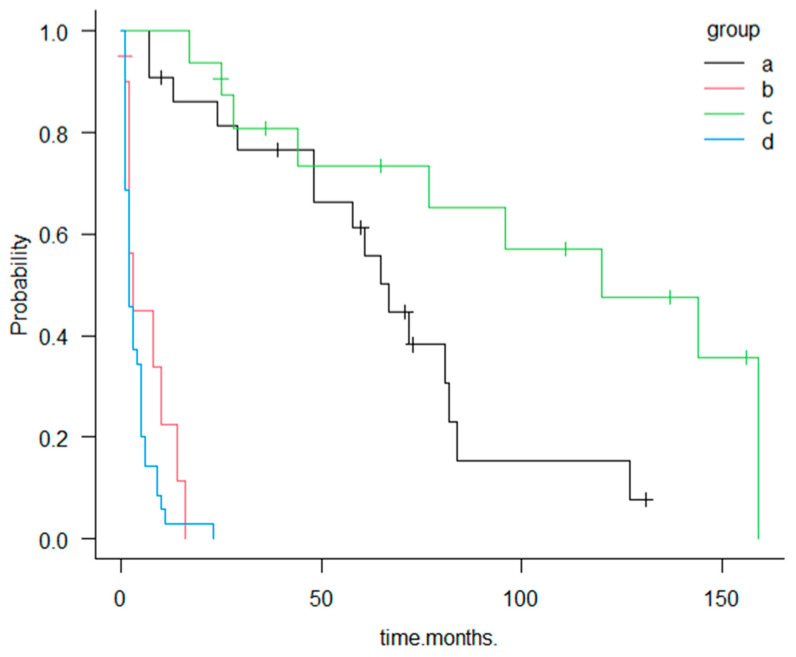
Kaplan–Meier survival curves for groups a–d. Group a: GBC with MPM (+) undergoing surgical resection; Group b: GBC MPM (+) without resection; Group c: GBC MPM (−) undergoing surgical resection; and Group d: GBC MPM (−) without resection. Resected cases (groups a and c) demonstrate significantly longer survival compared with unresected cases (groups b and d).

(4) When comparing tumor size and depth with stage between resected cases of both groups, we found the following distribution: Stage 0 (1/22 [4.5%] vs. 3/16 [18.8%]), Stage I (6/22 [27.3%] vs. 1/16 [6.3%]), Stage IIA (6/22 [27.3%] vs. 8/16 [50%]), Stage IIB (1/22 [4.5%] vs. 1/16 [6.3%]), Stage IIIA (1/22 [4.5%] vs. 0/16 [0%]), and Stage IIIB (3/22 [13.6%] vs. 3/16 [18.8%]). These results show no major differences in depth of invasion.
(5) A comparison of the MPM (+) resected and unresected groups in terms of first primary malignancy revealed similar trends: colorectal cancer (5/22 [22.7%] vs. 5/10 [50%]), gastric cancer (4/22 [18.2%] vs. 2/10 [20%]), and breast cancer (3/22 [13.6%] vs. 2/10 [20%]).
(6) Regarding prognosis, survival in unresected cases was short in both groups: 1–16 months with a mean of 5.9 months in the MPM (+) group and 1–9 months with a mean of 3.9 months in the MPM (−) group. Among resected cases, the mean survival was 49.5 months (7–131 months) in the MPM (+) group and 86.8 months (17–137 months) in the MPM (−) group. The longer survival observed in the MPM (−) group may be attributable to the larger proportion of long-term follow-up cases in this group relative to the MPM (+) group (Figure 3).

Most patients in the MPM (+) resected group (group a) were diagnosed with GBC while asymptomatic, either during postoperative surveillance or during evaluation for malignancies of other organs (21/22, 95%). These patients demonstrated markedly better survival than those in the symptomatic MPM (+) unresected group (group b), who were diagnosed only after symptom onset. The median survival was 67 months in group a compared with 3 months in group b, a difference that was both clinically substantial and statistically significant according to the log-rank test (*p* < 0.001).

In the Cox proportional hazards model, the symptomatic MPM (+) unresected group (group b) exhibited a markedly higher risk of mortality than the MPM (+) resected group (group a), with an estimated hazard ratio clearly exceeding 10, indicating a dramatically poorer prognosis.

Metachronous versus synchronous presentation

Patients with metachronous second primary GBC tended to have longer survival than those with synchronous presentation; however, this difference did not reach statistical significance in the log-rank test. Among the 22 resected MPM (+) GBC cases, postoperative survival in the 12 patients with synchronous GBC ranged from 7 to 84 months (mean, 49.2 months), whereas survival in the 10 patients with metachronous GBC ranged from 7 to 131 months (mean, 66.7 months). This trend was consistent with the clinical expectation that synchronous malignancies generally represent a greater overall oncologic burden than metachronous disease.

## 4. Discussion

GBC accounts for less than 2% of cancer-related deaths, but it is a very serious malignancy, with less than 10% of patients surviving for five years after diagnosis [1,2]. Roughly speaking, only 25% of patients undergo potentially curative surgery. Although only a relatively small proportion of patients ultimately undergo tumor resection, in this study, 68.7% of MPM (+) cases managed to undergo curative surgical resection. In contrast, only 31.3% of the MPM (−) cases were resected. The MPM (−) group likely reflects the typical clinical scenario. However, our findings indicate that a proper understanding of the concept of MPMs may substantially increase the resection rate for GBC.

GBC develops from the mucosal lining of the gallbladder lumen [2]. The early development of GBC is usually asymptomatic, and most patients are diagnosed at an advanced stage, at which point no curative treatment can be expected; thus, GBC is considered to be a highly lethal neoplasm.

The clinical symptoms of GBC include abdominal pain, jaundice, nausea and vomiting, and weight loss. These manifestations mainly result from biliary stenosis or obstruction caused by tumor progression, as well as from deterioration of one’s general condition [1,2]. In fact, in our present series, symptomatic cases accounted for 18.4% (7/38) of resected GBC cases (MPM (+) +MPM (−) cases), due to gallstone-related acute cholecystitis. The symptomatic cases were mainly MPM (−) (6/16: 37.5%) compared with MPM (+) (1/22: 4.5%). The prognosis was poor in both the MPM (+) cases (5.9 months) and MPM (−) cases (3.9 months) for the unresected groups. This finding reaffirms the traditional notion that a favorable prognosis for GBC cannot be expected unless the disease is detected at an early stage. Therefore, understanding the risk factors for GBC and identifying high-risk patients constitutes the most effective clinical strategy for its early detection.

High-risk factors associated with GBC occurrence include female sex [4], geographic location [5,12,24], congenital developmental anomalies [7,8,25], the presence of gallstones [5,6], and chronic inflammation of the gallbladder [1,2]. GBC exhibits a well-known global gender disparity, with an incidence in females that is three to six times higher than that in males [4]. It has long been suggested that female hormones play an important role in the etiology of GBC [4]. Numerous studies have identified multiparity as a significant risk factor for GBC, implying that the higher incidence in women may be related to reproductive factors. Hormonal effects and reproductive history—such as early or late menarche—are also considered to influence the development of GBC [4]. In contrast, some investigators have proposed that the female predominance of GBC simply reflects the markedly higher prevalence of gallstones among women [5,6]. In our study, however, the MPM (−) group showed a slight female predominance, whereas the MPM (+) group showed a slight male predominance—an observation that differs from previously reported trends and warrants attention. In addition, the incidence of GBC increases steadily with age, with more than two-thirds of patients being older than 65 years and the mean age at diagnosis being approximately 72 years. Several previous studies have documented that cancer risk rises with advancing age, primarily due to decreased immune function and other age-related biological changes [13,14,15]. In our study, the MPM (+) cases were slightly older (mean age, 72 years) than in typical GBC cohorts, suggesting that aging may have contributed to their development of multiple malignancies. As longevity is increasing in the general population, a higher incidence of MPMs is expected.

Regardless of sex differences, gallstones constitute one of the greatest risk factors for GBC, being present in 70–90% of cases [4,5]. Similarly to cholangitis, the persistent chronic inflammation of the gallbladder caused by gallstones is believed to promote carcinogenesis [7,8,25]. Primary sclerosing cholangitis (PSC) and chronic biliary inflammation associated with an anomalous pancreaticobiliary junction or choledochal cyst, which result in fibrosis and chronic inflammatory changes in the bile ducts, have also been linked to an increased risk of developing GBC [9]. A potential association between arsenic exposure due to natural contamination of groundwater and GBC incidence has been postulated for several years, in part due to the similarities between the geographic distribution of arsenic exposure and the incidence of GBC in some countries and regions [12]. Regarding environmental and dietary risk factors previously reported for GBC, the region in which our study was conducted does not correspond to areas known for such exposures; therefore, these factors are unlikely to have had any meaningful influence on our cohort.

Adenomas have long been considered precursor lesions for GBC due to the adenoma–adenocarcinoma sequence [26,27]. Papillary adenocarcinoma of the gallbladder has been reported as derived from intracholecystic papillary-tubular neoplasm (ICPNs) [28]. However, this possibility cannot be entirely excluded in this study, because all GBC cases were discovered at the stage of fully developed adenocarcinoma for most patients, prior gallbladder imaging was unavailable and gallbladder histology before the development of cancer was unknown.

Factors influencing the outcome of GBC are widely recognized to include the TNM stage and histological differentiation [2,29,30,31]. Radical tumor resection remains the only curative treatment for GBC, and the optimal extent of surgery and patient outcome depend on the tumor stage. According to the *AJCC Cancer Staging Manual, Eighth Edition*, T staging is classified as follows: Tis (carcinoma in situ), T1a (a carcinoma invading the lamina propria), T1b (a carcinoma invading the muscular layer), T2a (a carcinoma invading the perimuscular connective tissue on the peritoneal side, without involvement of the serosa), T2b (a tumor invading the perimuscular connective tissue on the hepatic side, with no extension into the liver), T3 (a carcinoma perforating the serosa and/or directly invading the liver), and T4 (a carcinoma invading the main portal vein or hepatic artery) [29,30]. It is prognostically important to distinguish between an early (muscle-confined) and an advanced (through the tunica muscularis) lesion. Generally, patients with early-stage GBC have a very favorable outcome (with a 10-year survival of 90%). Because the prognosis of GBC is closely associated with the T stage, the tumor should be resected as early as possible whenever feasible. In this study as well, approximately half of the cases were diagnosed at an advanced stage.

Multiple primary malignancies (MPMs) are defined as multiple tumors with different pathogenetic origins [13]. As for potential risk factors for MPM, viral infections, chemotherapy, and radiotherapy for the first primary malignancy have been implicated [13,14,15]. In our cases as well, these anti-cancer treatments and aging were presumed to be the major contributing factors. Malignancies can be synchronous or metachronous, occurring in multiple organs. Synchronous malignancies are usually defined as those appearing within 6 months from the diagnosis of a previous neoplasm, and metachronous neoplasms are defined as malignancies that appear more than 6 months after the first diagnosed neoplasm. It is vital to confirm the different degrees of primitiveness of the lesions, excluding a metastatic origin for one of them. Generally, the frequency and pattern of MPMs are thought to depend largely on the type of the first primary tumor and the duration of post-treatment follow-up. The general tendencies of MPMs include the following: (1) a higher occurrence in elderly patients and (2) a second primary cancer that usually occurs within 5 years after the first malignancy was diagnosed [14,15]. In previously published reports, combinations of gastrointestinal–gastrointestinal malignancies have most commonly involved gastric and colorectal cancers, although a few cases have included pancreatic or hepatic malignancies. In these combinations, synchronous tumors have consistently been associated with a poorer prognosis relative to metachronous tumors, presumably because they often represent advanced-stage cancers occurring simultaneously [16,17,18,19]. In contrast, combinations involving breast cancer—either breast–breast or breast cancer with malignancies of other organs—have generally been associated with more favorable outcomes [16,17,18,19]. To date, however, no comprehensive data are available regarding combinations involving GBC.

Between 2000 and 2024, to the best of our knowledge, only 28 cases of MPM involving the gallbladder were described [20,21,32,33,34,35,36,37,38,39,40,41,42,43,44,45,46,47,48,49,50,51,52,53,54]. According to the literature, metachronous neoplasms are much rarer than synchronous neoplasms (synchronous: 25; metachronous: 3) [20,21,32,33,34,35,36,37,38,39,40,41,42,43,44,45,46,47,48,49,50,51,52,53,54]. The majority of cases were synchronous tumors (25 cases), with a mean age of 68.0 years (range, 50–85 years) and a male-to-female ratio of 11:14. Among these cases, 21 involved gastrointestinal malignancies and 4 involved other types of tumor, a distribution that is largely consistent with the pattern observed in our cohort. There were no cases of hereditary syndromes, such as von Hippel–Lindau syndrome, that might have predisposed patients to the development of multiple neoplasms. Most of these publications describe GBC as the first primary tumor; cases in which GBC occurs as the second primary malignancy are even fewer, although the actual prevalence remains unclear.

In our series, gastrointestinal cancer constituted the majority of the first primary tumors in the MPM (+) GBC groups, as in previous reports where gastrointestinal cancer was also the most common first primary malignancy [14,15,55]. In both the literature and our cases, this combination was predominant among synchronous tumors. These findings suggest that the combination of gastrointestinal cancer and GBC is particularly common. Therefore, in patients diagnosed with GBC, a thorough search for synchronous or prior gastrointestinal malignancies is warranted. Conversely, for patients with gastrointestinal cancers, careful evaluation of the gallbladder should be performed.

However, a substantial discrepancy was observed between our metachronous cases and those previously reported. In the three previously published metachronous cases, the first primary tumor was typically GBC, and the second primary tumor was most often pancreatic cancer [33,44,52]. In contrast, in our series, the majority of metachronous cases involved gastrointestinal cancers as the first primary tumor and GBC as the second primary malignancy. This discrepancy likely reflects the extremely small number of metachronous cases reported as data and considerable variability in the intervals between the first and second primary cancers. Further accumulation of similar cases will be necessary. At the very least, during follow-ups after treatment of gastrointestinal malignancies, systemic surveillance of the gallbladder is advisable.

Regarding diagnostic strategies, because imaging findings may vary depending on the institution in question and lesion size, it is difficult to make generalized statements; however, ultrasonography has traditionally been regarded as the most suitable modality for the detection and characterization of small gallbladder lesions [23,56,57,58]. At our institution, whole-body CT is routinely performed both before treatment for cancer patients and during scheduled follow-ups after therapy. This practice is widely accepted. When an abnormality of the gallbladder wall is detected on CT, CEUS is subsequently performed to assess the qualitative nature of the gallbladder lesion. This approach is considered rational; however, it requires a facility capable of maintaining a high level of CEUS expertise and thus cannot be universally recommended. Ultimately, at institutions where CEUS is feasible, the combination of US and CEUS is likely to impose the least burden on patients, whereas endoscopic ultrasonography (EUS) and MRI may be reserved for cases in which US/CEUS findings remain inconclusive [22,23,59,60]. Given the variation in proficiency across institutions, the optimal diagnostic system should be determined individually by each facility.

Regarding follow-ups after treatment of the first primary malignancy, a whole-body CT every six months is generally considered standard practice. However, two important questions remain unanswered: (1) Should additional imaging modalities be incorporated, and if so, what is the rationale for doing so? (2) What is the appropriate duration of surveillance? At our institution, surveillance is usually performed using chest/abdomen/pelvis CT at intervals of 3–6 months, with CEUS added when suspicious findings are noted on a follow-up CT. Nevertheless, this strategy may require modification as more metachronous cases accumulate in the future.

This study has several limitations. First, there were relatively few cases. This limitation became even more evident when the cases were further subdivided into four groups. Nevertheless, several meaningful conclusions could still be drawn. Second, although major risk factors for gallbladder carcinoma, such as gallstones and sex differences, were analyzable, data on other minor risk factors—namely, diabetes mellitus, obesity, and dietary habits—were not available in the patient records. Therefore, these factors could not be accurately evaluated or excluded. Third, this investigation was conducted exclusively in a Japanese population. Whether these findings can be generalized beyond ethnic and geographic boundaries remains uncertain, and future multinational comparative studies will be necessary to clarify this issue. Fourth, the evaluation of contrast-enhanced ultrasonography (CEUS) was performed using a limited number of ultrasound systems. However, given that no major differences in CEUS enhancement patterns have been reported among currently available ultrasound devices, it is unlikely that our findings would differ substantially from those obtained in future studies employing other systems.

## 5. Conclusions

This study provides clinically relevant insights into the association between MPMs and GBC, a relationship that has only been sparsely documented to date. Although based on a relatively limited number of cases, our analysis demonstrates that GBC in MPM (+) cases most commonly occurred as a second primary malignancy, frequently following gastrointestinal cancers and often in the absence of classical risk factors such as gallstones.

Importantly, MPM (+) cases who were diagnosed before the onset of symptoms showed a markedly better prognosis than those diagnosed after symptom development, highlighting the critical role of regular post-treatment surveillance. In particular, the comparison between resected and unresected MPM (+) cases suggests that systematic follow-ups—including careful evaluation of the gallbladder—can facilitate detection at an earlier, resectable stage, thereby substantially improving outcomes.

From a diagnostic perspective, our findings support the use of ultrasonography combined with CEUS as an effective and minimally invasive strategy for the evaluation of gallbladder lesions detected during follow-up imaging. While the optimal surveillance protocol may vary among institutions depending on available expertise and resources, heightened awareness of MPM-related GBC and focused gallbladder assessment during follow-up of gastrointestinal malignancies appear warranted.

In conclusion, recognizing MPMs as a clinical context associated with potentially resectable GBC may contribute to earlier detection and improved prognosis. Further large-scale, multicenter studies are needed to validate these findings and establish evidence-based surveillance strategies for patients at risk.

## Figures and Tables

**Table 1:** Clinical features and outcomes of 83 gallbladder cancer cases according to multiple primary malignancy status and resectability.

	MPM (+), Resected	MPM (+), Unresected	MPM (−), Resected	MPM (−), Unresected
No. of patients	22	10	16	35
Age, years (mean)	59–88 (75.3)	79–96 (86.4)	51–86 (73.7)	58–91 (79.2)
Sex (male/female)	12/10	6/4	8/8	9/26
Synchronous/metachronous	12/10	5/5	–	–
First primary malignancy, n (%)				
Gastrointestinal cancer	14 (64)	8 (80)	–	–
Non-gastrointestinal cancer	8 (36)	2 (20)	–	–
Major risk factors for GBC				
Gallstones, n (%)	2 (9)	1 (10)	6 (38)	29 (82)
Tumor diameter, mm (mean)	26.3	–	22.2	–
Stage ≤ IIB, n (%)	17 (77)	0 (0)	13 (81)	0 (0)
Survival time, months (mean)	57.1	5.9	87.3	3.9

GBC, gallbladder cancer; MPM, multiple primary malignancy; MPM (+), resected group mainly consisted of patients under regular surveillance after treatment of the first primary malignancy; MPM (−), unresected group included patients without regular follow-up, in whom GBC is detected at an advanced stage; GI cancers include colorectal, gastric, esophageal, pancreatic and bile duct cancers. Stage classification is based on the UICC/AJCC TNM staging system (8th edition).

## Data Availability

The original contributions presented in this study are included in the article. Further inquiries can be directed to the corresponding author.

## Abbreviations

The following abbreviations are used in this manuscript:

GBC	Gallbladder cancer
PBM	Pancreaticobiliary maljunction
MPMs	Multiple primary malignancies
MPM (+)	Multiple primary malignancy-positive
MPM (−)	Multiple primary malignancy-negative
US	Ultrasound
CEUS	Contrast-enhanced ultrasound
CT	Computed tomography
MRI	Magnetic resonance imaging
